# Mitochondrial DNA Haplogroup Related to the Prevalence of *Helicobacter pylori*

**DOI:** 10.3390/cells10092482

**Published:** 2021-09-19

**Authors:** Yeonmi Lee, Sun-Mi Lee, Jiwan Choi, Seoon Kang, Seongjun So, Deokhoon Kim, Ji-Yong Ahn, Hwoon-Yong Jung, Jin-Yong Jeong, Eunju Kang

**Affiliations:** 1 Department of Biomedical Science, College of Life Science and Center for Embryo and Stem Cell Research, CHA Advanced Research Institute, CHA University, Seongnam, Gyeonggi 13488, Korea; yeonmilee82@chamc.co.kr (Y.L.); jiwanc624@gmail.com (J.C.); luceamso@gmail.com (S.K.); soseongjun7@gmail.com (S.S.); 2 Asan Medical Center, Asan Institute for Life Sciences, Seoul 05505, Korea; eclipse-lsm@hanmail.net; 3 Department of Convergence Medicine, Asan Medical Center, University of Ulsan College of Medicine, Seoul 05505, Korea; 4 Department of Pathology, Asan Medical Center, University of Ulsan College of Medicine, Seoul 05505, Korea; coonya@gmail.com; 5 Department of Gastroenterology, Asan Medical Center, University of Ulsan College of Medicine, Seoul 05505, Korea; ji110@hanmail.net

**Keywords:** *Helicobacter pylori*, mitochondrial genome, mutation, haplogroup, susceptibility

## Abstract

Mitochondria are essential organelles that are not only responsible for energy production but are also involved in cell metabolism, calcium homeostasis, and apoptosis. Targeting mitochondria is a key strategy for bacteria to subvert host cells’ physiology and promote infection. *Helicobacter (H.) pylori* targets mitochondria directly. However, mitochondrial genome (mtDNA) polymorphism (haplogroup) is not yet considered an important factor for *H. pylori* infection. Here, we clarified the association of mitochondrial haplogroups with *H. pylori* prevalence and the ability to perform damage. Seven mtDNA haplogroups were identified among 28 *H. pylori*-positive subjects. Haplogroup B was present at a higher frequency and haplotype D at a lower one in the *H. pylori* population than in that of the *H. pylori*-negative one. The fibroblasts carrying high-frequency haplogroup displayed a higher apoptotic rate and diminished mitochondrial respiration following *H. pylori* infection. mtDNA mutations were accumulated more in the *H. pylori*-positive population than in that of the *H. pylori*-negative one in old age. Among the mutations, 57% were located in RNA genes or nonsynonymous protein-coding regions in the *H. pylori*-positive population, while 35% were in the *H. pylori*-negative one. We concluded that gastric disease caused by *Helicobacter* virulence could be associated with haplogroups and mtDNA mutations.

## 1. Introduction

*Helicobacter pylori* is a gram-negative, microaerophilic bacterium that colonizes the gastric mucosa of at least half of all humans worldwide [1]. The integrity of the gastric mucosa is maintained by a balance between cell proliferation and death. However, *H. pylori* can induce their imbalance, resulting in gastric diseases, such as chronic gastritis, peptic ulceration, gastric carcinoma, and lymphoma [2,3]. Although the prevalence of *H. pylori* in the global population was steadily decreasing, it was recently estimated to be approximately 50% [4,5,6].

Mitochondria play essential roles in normal cellular function in eukaryotes, such as energy production, calcium homeostasis, apoptotic activation, and cell death [7,8]. Several studies showed that *H. pylori* induces the instability of mtDNA in stomach tissue [9,10]. Indeed, mtDNA mutations were identified in chronic gastritis, peptic ulcer tissues, and gastric cancer in the presence of *H. pylori*. Polymorphisms of the host nuclear genome were demonstrated to be associated with the prevalence of *H. pylori* and related diseases [11,12,13]. These polymorphisms may regulate pro- and anti-inflammatory processes, and affect the expression levels of inflammatory mediators, increasing *H. pylori* activity [13]. Furthermore, different nuclear gene polymorphisms can influence not only the susceptibility to *H. pylori* infection, but also the response to treatment [14].

Mitochondrial haplogroups are collections of similar haplotypes defined by combinations of single-nucleotide polymorphisms in mtDNA inherited from the mother [15]. Specific mitochondrial haplogroups are also closely linked to the risk of some diseases, such as Alzheimer’s disease with haplogroup U, age-related macular degeneration with the JTU haplogroup cluster, and type 2 diabetes with haplogroup N9a [16,17,18,19]. However, to the best of our knowledge, no reports were published on the association between specific mtDNA haplogroups and *H. pylori*.

Here, we determined mtDNA haplogroup in each individual and investigated mtDNA mutations in gastric tissue infected with *H. pylori*. We also measured mitochondrial respiratory function and apoptosis in the cultured fibroblasts with selected haplogroups.

## 2. Materials and Methods

### 2.1. Subjects and Sample Collection

All subjects were recruited from Asan Medical Center, Seoul, Korea. Written informed consent was obtained from each subject. Two mucosal biopsy specimens from 43 subjects, 45–78-year-old of Korean people, were obtained from the gastric antrum and corpus greater curvature using standard-sized biopsy forceps during endoscopy. The *H. pylori* test was performed on both the antrum and body because the *H. pylori* positivity rate in the antrum and body may be different depending on the patient’s gastric acid secretion level. The positive or negative of *H. pylori* in mucosal biopsy specimens were confirmed by culturing on Brucella broth agar. This study was approved by the Institutional Review Board for Human Research at Asan Medical Center (IRB number: 2020-0108).

### 2.2. Preparation of H. pylori

Cultured fibroblasts were infected with *H. pylori* strain ATCC 43504 (ATCC, Manassas, VA, USA) [20]. *H. pylori* strains were cultured on Brucella broth agar, which was supplemented with 5% sheep blood and vancomycin (10 µg/mL), trimethoprim (5 µg/mL), amphotericin B (5 µg/mL), and polymyxin B (2.5 IU). The *H. pylori* strains were cultured under microaerophilic conditions (5% O_2_, 10% CO_2_, 85% N_2_). All stock was maintained at −70 °C in Brucella broth supplemented with 15% glycerol.

### 2.3. mtDNA Sequencing by Miseq

Biopsied gastric tissues were collected during endoscopy in patients with gastric disease and extracted total genomic DNA using Gentra DNA Extraction Kit (QIAGEN, Hilden, North Rhine-Westphalia, German) in accordance with the manufacturer’s protocol. The whole mtDNA was amplified using TaKaRa LA Taq (TaKaRa, Kusatsu, Shiga, Japan), and mtDNA sequencing was performed using Miseq (Illumina, San Diego, CA, USA) as previously described, with minor modification [21]. Briefly, whole mtDNA amplification was performed using a two-fragment PCR reaction employing the following primers: fragment 1: F-3163 GCCTTCCCCCGTAAATGATA and R-11599 TGTTTGTCGTAGGCAGATGG; fragment 2: F-11506 TCTCAACCCCCTGACAAAAC and R-3259 TATGCGATTACCGGGCTCT. PCR reaction was performed under the following conditions: one cycle at 94 °C for 5 min; 35 cycles at 94 °C for 20 s, 56 °C for 20 s, and 68 °C for 8 min, followed by one cycle at 68 °C for 3 min with TaKaRa LA Taq (TaKaRa). The concentration of PCR products was measured using a Qubit 2.0 Fluorometer (Invitrogen, Waltham, MA, USA). Library preparation was performed using a Nextera XT DNA sample preparation kits (Illumina) following the manufacturer’s manual. Sequencing was performed on the Illumina MiSeq platform, approximately 5 Mb per sample, and the data were analyzed using NextGENe software. The whole mtDNA size is 16,569 bp, much less than the size of exome or whole nuclear genome, therefore, the Miseq data size of mtDNA is Mb level, not Gb level like whole nuclear or exome analysis. Briefly, sequence reads ranging from 250 to 500 bp were quality-filtered and processed using NextGENe software and an algorithm similar to BLAT. The sequence error correction feature (condensation) was performed to reduce false-positive variants and produce a sample consensus sequence and variant calls for each sample. Alignment without sequence condensation was used to calculate the percentage of the mitochondrial genome with a depth of coverage of 1000. Starting from quality FASTQ reads, the reads were quality-filtered and converted to FASTA format. Filtered reads were aligned to the human mitochondrial sequence reference NC_012920.1, followed by variant calling. Variant heteroplasmy was calculated using NextGENe software as follows: base heteroplasmy (mutant allele frequency %) = mutant allele (forward + reverse)/total coverage of all alleles C, G, T, and A (forward + reverse) × 100. Variants that were not validated were removed from the results. mtDNA haplogroup of each subject was determined using the human mitochondrial genome database, and MitoMAP (https://www.mitomap.org, accessed on 25 February 2021) and homoplasmic variants were used to establish haplogroup. The locus of variants in mtDNA and GB frequency of each variant (frequency of variants reported in GenBank) are also available in Supplementary Tables S1 and S2.

### 2.4. Infection of Cultured Fibroblasts with H. pylori

Fibroblasts, which were already analyzed for mtDNA haplogroups for an independent mitochondrial project, were used to study apoptosis and mitochondrial function. Approximately 5 × 10^4^ cells were plated onto a 6-well cell culture plate and culture in F12/DMEM with 10% FBS (Hyclone, GE Healthcare Life Sciences, Logan, UT, USA), 100 units/mL penicillin and 100 μg/mL streptomycin (Hyclone), 100 μM β-mercaptoethanol (Sigma, St. Louis, MO, USA), and 100 μM nonessential amino acids (Gibco, Life Technologies Corporation, Grand Island, NY, USA) under 5% CO^2^ at 37 °C in a humidified incubator for 5 days. The media were changed every 2 days. On day 5 of cell culture, bacterial cells were added to fibroblasts at a multiplicity of infection (MOI) of 50–300 bacteria for total *H. pylori*. For 24 h of *H. pylori* infection, skin fibroblasts were washed three times with PBS and incubated in an antibiotic-free medium. Cultures were maintained at 37 °C under an atmosphere of 10% CO_2_.

### 2.5. Assessment of Apoptosis

Apoptosis assay was performed 24 h after the completion of *H. pylori* infection using the Dead Cell Apoptosis Kit (Invitrogen), in accordance with the manufacturer’s protocol. Briefly, harvested cells (~1 × 10^6^ cells/mL) were incubated in annexin-binding buffer mixed with Alexa Fluor^®^ 488 annexin V and PI solution for 15 min at room temperature. After the incubation period, stained cells were analyzed by flow cytometry, measuring the fluorescence emission at 530 nm and 575 nm using 488 nm excitation.

### 2.6. Assessment of Mitochondrial Respiratory Functions

Mitochondrial respiratory function was measured 24 h after the completion of *H. pylori* infection using the XF Cell Mito Stress Test Kit in an XF24 Extracellular Flux Analyzer (Seahorse Biosciences, North Billerica, MA, USA), as previously described [21]. The mitochondrial oxygen consumption rate (OCR) was measured by the serial addition of oligomycin (2 µM) for ATP production (oligomycin OCR basal OCR), carbonyl cyanide 4-(trifluoromethoxy) phenylhydrazone (FCCP, 1 µM) for maximal respiration, and spare respiratory capacity (maximal OCR basal OCR), antimycin A (0.5 µM), and rotenone (0.5 µM) for nonmitochondrial oxygen usage. The value was normalized to baseline oxygen consumption with 1 ng of DNA.

### 2.7. Statistics

Assessment of mitochondrial respiratory functions was triplicated, and assessment of apoptosis was repeated four times. The data are presented as mean ± SEM or SD. *p* < 0.05 was considered significant. Data were analyzed using regression analysis, independent-group *t*-test, or Fisher’s exact tests for two-group comparisons and ANOVA with Tukey for multiple group comparisons (GraphPad Prism).

## 3. Results

### 3.1. Relationship between mtDNA Haplogroups and H. pylori Prevalence

**Figure 1:**
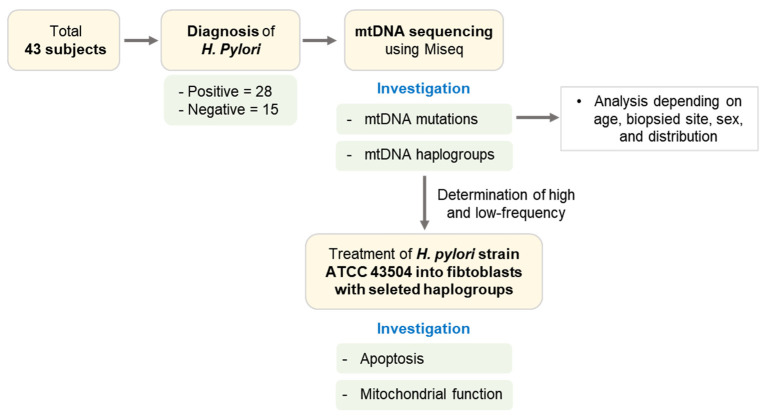
Experimental design of study. A total of 43 subjects, *H. pylori*-positive (*n* = 28), -negative population (*n* = 15), were investigated for mtDNA mutations and haplogroups. High- and low-frequency haplogroups in *H. Pylori*-positive population were determined, and fibroblasts with selected haplogroups were infected with *H. Pylori* strain ATCC 43504. Apoptosis and mitochondrial function were investigated in infected fibroblasts with selected haplogroups. mtDNA mutations were analyzed depending on age, biopsied site, sex, and distribution on mDNA location.

mtDNA haplogroups were determined based on the human mitochondrial genome database, MitoMAP (https://www.mitomap.org, accessed on 25 February 2021), with homoplasmic variants in Miseq sequencing results. A total of 43 subjects, *H. pylori*-positive (*n* = 28), -negative population (*n* = 15), were investigated their mtDNA haplogroups and mutations. Further, the apoptosis and mitochondrial dysfunction due to *H. pylori* infection were evaluated in fibroblasts with selected haplogroups (Figure 1).

Firstly, we investigated whether particular haplogroups of mtDNA were associated with *H. pylori* infection. The frequencies of mtDNA haplogroups were analyzed in *H. pylori*-negative (*n* = 15), -positive populations (*n* = 28), and the Korean population (Table 1).

**Figure 2:**
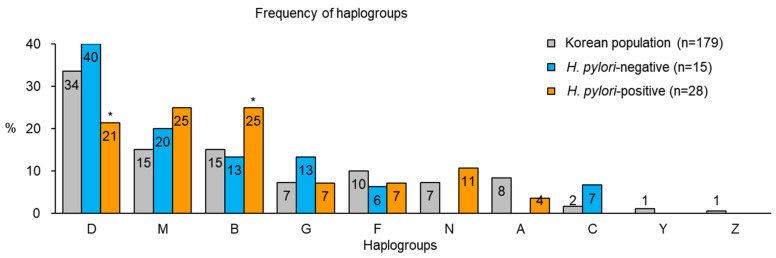
Frequencies of mtDNA haplogroups in Korean population and *H. pylori*-negative and -positive populations. *H. pylori*-positive population showed a significantly higher frequency of haplogroup B and a significantly lower frequency of haplogroup D than that of the *H. pylori*-negative population and the Korean population (* *p* < 0.05).

Ten haplogroups were identified in 179 subjects in the Korean population [22], while the *H. pylori*-negative and -positive populations included 6 and 7 haplogroups in 15 and 28 subjects, respectively (Figure 2). Among the haplogroups, haplogroup D showed a significantly lower rate (*p* < 0.05) in the *H. pylori*-positive population than in that of the Korean population and the *H. pylori*-negative population (21% vs. 34% and 40%, respectively), whereas haplogroup B showed a significantly higher proportion (*p* < 0.05, 25% vs. 15% and 13%, respectively). The remaining haplogroup displayed no significant difference in the frequency among the groups (Figure 2).

Based on these results, we demonstrated that the frequency of haplogroup B was higher in the *H. pylori*-positive population, suggesting that this haplogroup could be associated with a greater risk of *H. pylori* infection.

### 3.2. Validation of Susceptibility to H. pylori Infection Associated with mtDNA Haplogroup Prevalence

**Figure 3:**
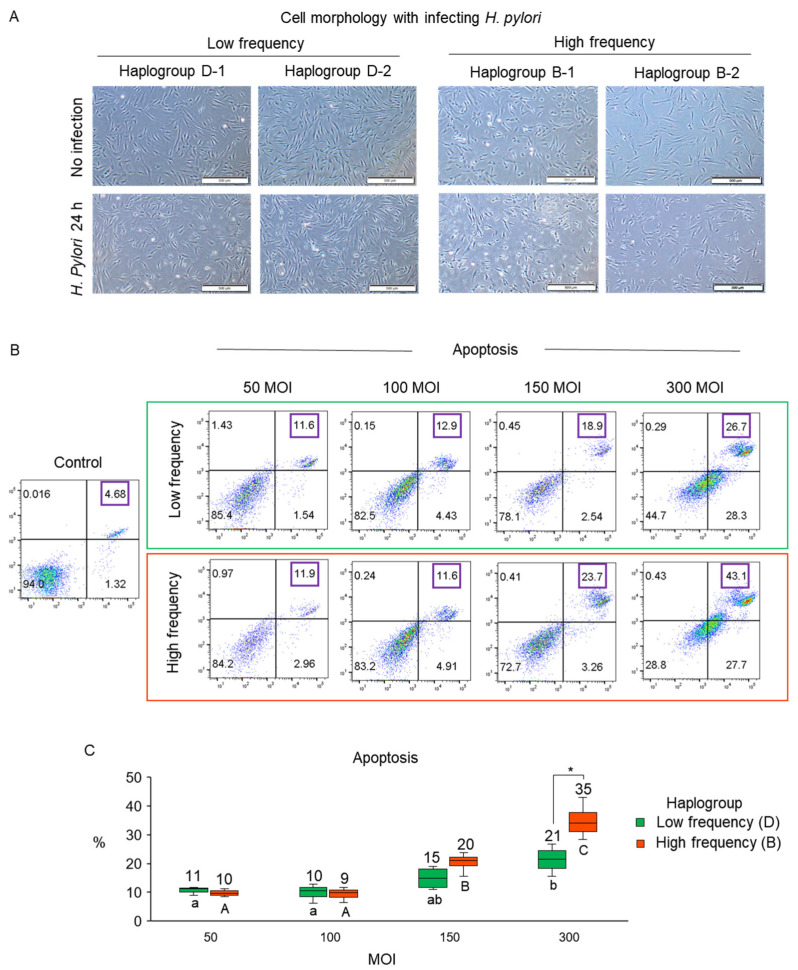
Different apoptosis rates upon *H. pylori* infection depending on mtDNA haplogroups. (**A**) Different cell morphology before and after *H.* *pylori* infection depending on the mtDNA haplogroup. High-frequency haplogroup showed shrinkage-like morphology. Scale bar: 500 μm. (**B**) Apoptosis rates in low- and high-frequency haplogroups with *H. pylori* infection at various MOI. Purple boxes indicate apoptotic rate. (**C**) Increased apoptosis in high-frequency haplogroup upon *H. pylori* infection at high MOI. Letters a and b indicate significant differences among different MOI treatments in low-frequency haplogroup (*p* < 0.05). Letter A, B, and C indicate significant differences among different MOI treatments in high-frequency haplogroup (*p* < 0.05). * indicates a significant difference between low and high-frequency haplogroup at 300 MOI (*p* < 0.05).

Based on a previous study that *H. pylori* components affect apoptosis and oxidative stress in fibroblasts [23], we infected fibroblasts with individual haplogroups with *H. pylori* strain ATCC 43504, as CagA and VacA-positive strain, which could induce gastric cell dysfunction and metabolite alterations [24,25,26]. Then, apoptosis and mitochondrial respiratory function were analyzed to determine whether specific haplogroups of mtDNA were associated with *H. pylori*. Fibroblasts with high- (haplogroup B) and low-frequency haplogroups (haplogroup D) were infected with *H. pylori* with an MOI of 50, 100, 150, and 300 for 24 h (Figure 2). We observed the cell morphology after *H. pylori* infection compared to that of noninfected cells. The cell shrinkage, which is usually observed in apoptotic cells [27], was more frequently observed in fibroblasts with high-frequency haplogroup than in that of low-frequency haplogroup at MOI 300 (Figure 3A).

The morphology of the fibroblasts with the high-frequency haplogroup was changed at MOI 300 compared to noninfected cells. Upon *H. pylori* infection at 50, 100, and 150 MOI, the rates of apoptosis were similar between the high- and low-frequency haplogroups. However, on *H. pylori* infection at 300 MOI, there was significantly increased apoptosis in high-frequency haplogroup than in that of the low-frequency haplogroup (mean apoptotic rates: 35% vs. 21% for high- and low-frequency haplogroup, respectively; Figure 3B). Both haplogroups showed significantly higher apoptosis at 300 MOI compared to that of 50 and 150 MOI, while only high-frequency haplogroup displayed significantly higher apoptosis at 150 MOIs than that of 50 and 100 MOIs, and 300 MOIs than that of 150 MOIs (Figure 3C). Taking these findings together, we demonstrated that the cell damage caused by *H. pylori* can be associated with mtDNA haplogroups.

Next, we investigated the degree of mitochondrial respiration of mtDNA haplogroups (haplogroup B for high susceptibility; haplogroup D for low susceptibility) following *H. pylori* infection. Initially, the cells were infected with *H. pylori* at 300 MOI, which induced the most apoptosis in the high-susceptibility haplogroup.

**Figure 4:**
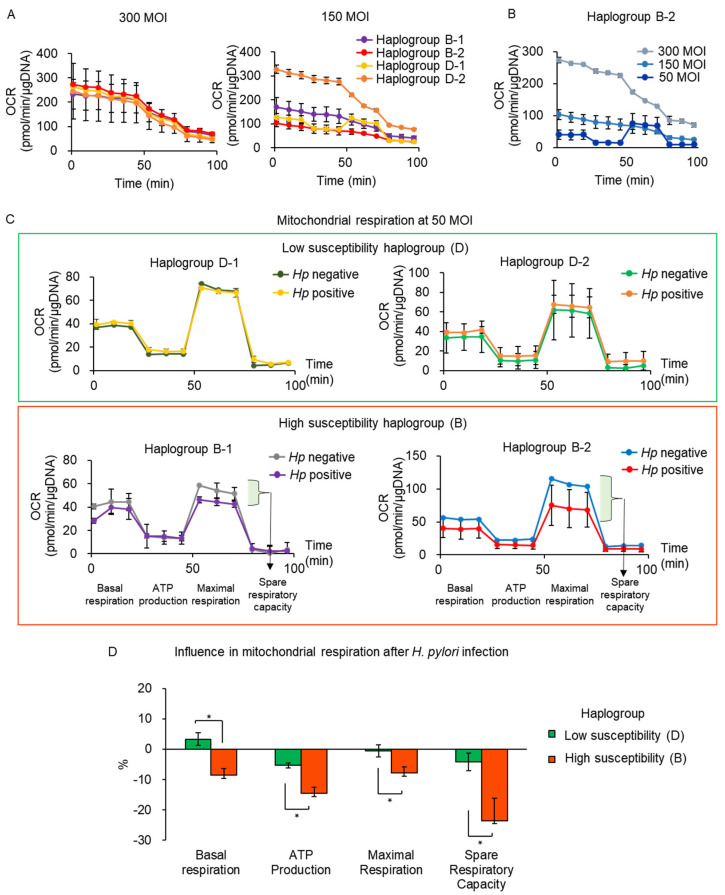
Decreased mitochondrial respiration in high-susceptibility haplogroup by *H. pylori* infection. (**A**) Abnormal assessment of mitochondrial respiration with high MOI of *H. pylori* infection using seahorse assay. (**B**) Comparison of mitochondrial respiration assessment upon infection with *H. pylori* of haplogroup B-2 with that of various MOI. *H. pylori* infection at low MOI (50) enabled normal assessment of mitochondrial respiration. (**C**) Mitochondrial respiration measurement upon *H. pylori* infection at 50 MOIs. OCR was decreased in the high-susceptibility haplogroup (higher frequency in the *H. pylori*-positive population than in the negative one) after *H. pylori* infection. Low-susceptibility haplogroup (lower frequency in the *H. pylori*-positive population) revealed similar OCR after *H. pylori* infection. (**D**) Influence of *H. pylori* infection on mitochondrial respiration. Mitochondrial respiration was significantly decreased in high-susceptibility haplogroup after *H. pylori* infection than in that of low-susceptibility haplogroup (* *p* < 0.05). Error bars indicate mean ± SD (**A**–**C**) or mean ± SEM (**D**).

As shown in Figure 4A, OCR, an indicator of mitochondrial respiration and energy production, was measured abnormally upon *H. pylori* infection at 300 MOI, which possibly resulted from severe mitochondrial dysfunction under *H. pylori*. Therefore, we performed *H. pylori* infection at a reduced MOI to induce less mitochondrial damage in fibroblasts. When the cells were infected with *H. pylori* at 150 MOIs, most of them still showed abnormal OCR measurements (Figure 4A). We then decreased the MOI to 50 in fibroblasts of haplogroup B-2, upon which OCR was measured normally and mitochondrial respiration could be properly analyzed (Figure 4B). Therefore, we set the MOI of *H. pylori* to 50 for the evaluation of mitochondrial respiration. *H. pylori*-infected cells exhibited lower OCR than uninfected cells in the high-susceptibility haplogroup (Figure 4C). However, the low-susceptibility haplogroup showed similar OCR between *H. pylori*-infected and uninfected cells (Figure 4C).

We calculated the rate of change in mitochondrial respiration after *H. pylori* infection and compared the findings between the high- and low-susceptibility haplogroups (Figure 4D). The low-susceptibility haplogroup showed changes under 5% after *H. pylori* infection. Meanwhile, the high-susceptibility haplogroup showed 9%, 15%, 9%, and 24% decreases in basal respiration, ATP production, maximal respiration, and spare respiratory capacity after *H. pylori* infection, respectively, which were more significant decreases than the low-susceptibility haplogroup (Figure 4D). These results indicated that the high-susceptibility haplogroup showed decreased mitochondrial respiration compared with that of the low-susceptibility haplogroup, suggesting the decrease in metabolic function by *H. pylori* infection in the high-susceptibility haplogroup.

### 3.3. mtDNA Mutations with H. pylori Infection

**Figure 5:**
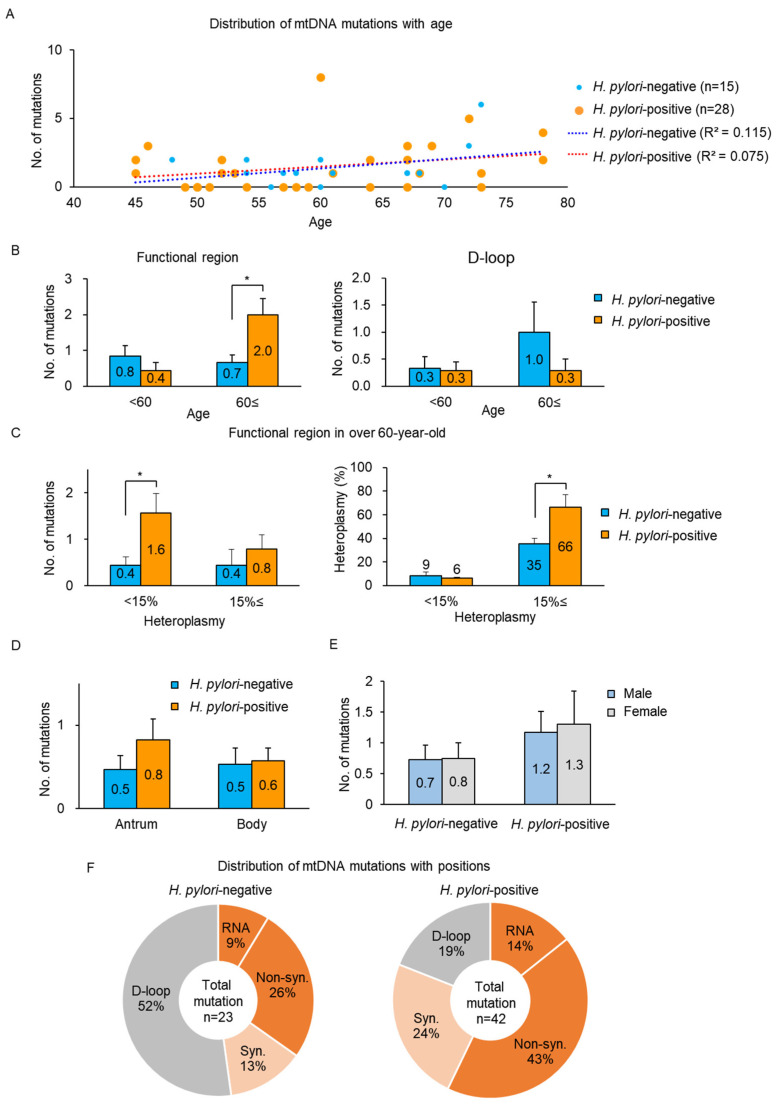
mtDNA mutations detected in *H. pylori*-negative and -positive populations. (**A**) Distribution of mtDNA mutations with age in *H. pylori*-negative (*n* = 15) and -positive populations (*n* = 28). There was no significant difference between *H. pylori*-negative and positive populations when analyzed by regression analysis. R^2^ means Pearson’s r values. (**B**) There was a significant increase in number of mtDNA mutations in functional region at the age of 60 years or older in the *H. pylori*-positive population (* *p* < 0.05). D-loop showed similar numbers between *H. pylori*-positive and -negative populations (*H. pylori*-positive: <60, *n* = 14 and 60≤, *n* = 14; *H. pylori*-negative: <60, *n* = 6 and 60≤, *n* = 9). (**C**) Increased number and heteroplasmy of mtDNA mutations at age of 60 years or older in *H. pylori*-positive population. Number of mtDNA mutations under 15% heteroplasmy and heteroplasmy of mtDNA mutations above 15% heteroplasmy were significantly increased (* *p* < 0.05) in *H. pylori*-positive population (*H. pylori*-positive: *n* = 14; *H. pylori*-negative: *n* = 9). (**D**) mtDNA mutations in functional regions preferentially occurred in antrum of the stomach of *H. pylori*-positive population. Body had similar numbers of mtDNA mutations in *H. pylori*-negative and -positive populations (*H. pylori*-positive: *n* = 28; *H. pylori*-negative: *n* = 15). (**E**) Comparison of mtDNA mutations in males and females. Males and females had similar numbers of mtDNA mutations in functional regions in *H. pylori*-positive population (*H. pylori*-positive: male, *n* = 18 and female, *n* = 10; *H. pylori*-negative: male, *n* = 11 and female, *n* = 4). (**F**) Distribution of mtDNA mutations in *H. pylori*-negative (*n* = 15) and -positive populations (*n* = 28). More mtDNA mutations were nonsynonymous substitutions and located in RNA genes in the *H. pylori*-positive population compared with that of -negative population. Error bars indicate mean ± SEM.

mtDNA mutations in gastric tissue DNA from 28 *H. pylori*-positive and 15 *H. pylori*-negative subjects (Table 2) were analyzed using Illumina MiSeq-based mtDNA sequencing. There was no significant difference in the average age depending on the presence of *H. pylori* infection or sex (Table 2). Among 43 subjects, 29 subjects harbored 1–8 mtDNA mutations with various levels of heteroplasmy (2–97%) or homoplasmy in individual gastric tissue (Figure 5A and Supplementary Table S3), and there was no significant difference between *H. pylori*-positive (*n* = 28) and *H. pylori*-negative subjects (*n* = 15). The number of mtDNA mutations in functional regions (coding regions and RNAs) was significantly higher (*p* < 0.05) in the *H. pylori*-positive population than in that of the -negative population at the age of 60 years or older, while similar rates were found in the D-loop as a noncoding region (Figure 5B). Under the age of 60, the numbers of mtDNA mutations in functional regions and D-loop were comparable between *H. pylori*-positive and -negative populations. In the case of mtDNA mutations in functional regions at the age of 60 years or older, the number of mtDNA mutations under 15% heteroplasmy was significantly higher (*p* < 0.05) in the *H. pylori*-positive population than in that of the negative one, and the mean heteroplasmy of mtDNA mutations above 15% heteroplasmy was significantly higher in the *H. pylori*-positive population (*p* < 0.05, Figure 5C).

We investigated whether mtDNA mutations were associated with specific sites in gastric tissue (antrum and body) or sex in the *H. pylori*-positive population. The mtDNA mutations were detected slightly more in the antrum in the *H. pylori*-positive group, but the body showed similarities between *H. pylori*-positive and -negative populations (Figure 5D). Males and females displayed comparable numbers of mtDNA mutations in the *H. pylori*-positive population (Figure 5E).

Among 42 mutations in the *H. pylori*-positive population, 43% were nonsynonymous substitutions in the coding regions of mtDNA proteins, resulting in amino acid changes (Figure 5F). 14% were in tRNA or rRNA genes that were associated with oxidative phosphorylation (OXPHOS) [21]. Meanwhile, in the *H. pylori*-negative population, half of the mutations (52%) was detected in the D-loop region as a noncoding region, and only 26% and 9% of mutations were nonsynonymous substitutions and located in RNA genes, respectively.

**Figure 6:**
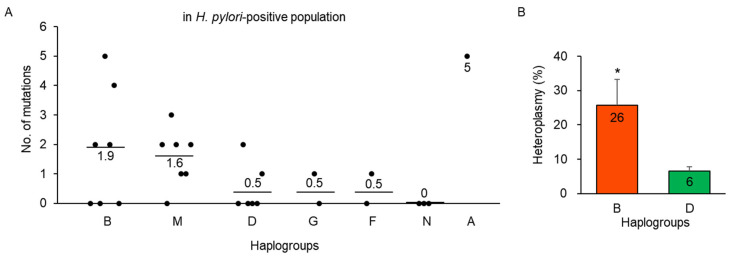
mtDNA mutations in each haplogroup of the *H. pylori*-positive populations. (**A**) Number of mtDNA mutations of functional regions in the *H. pylori*-positive population. Haplogroup B was found to have a higher number of mtDNA mutations than that of haplogroup D, but the difference was not significant. (**B**) Increased heteroplasmy in functional regions in the *H. pylori*-positive population. Average heteroplasmy of mtDNA mutations was significantly higher in haplogroup B than in that of haplogroup D (* *p* < 0.05).

Additionally, we analyzed mtDNA mutations based on each haplogroup within the *H. pylori*-positive population. We focused on haplogroups D and B because of the significant changes in their frequency after *H. pylori* infection (Figure 2). Haplogroup B was found to have a higher number of mtDNA mutations in the functional region than haplogroup D, but the difference did not reach significance (Figure 6A). However, the average heteroplasmy of mtDNA mutations was significantly higher *(p* < 0.05) in haplogroup B than in that of haplogroup D (Figure 6B).

In summary, mtDNA mutations were particularly accumulated in the *H. pylori*-positive population at the age of 60 years or older, and they were frequently distributed in functional regions in the *H. pylori*-positive population compared with the findings in the *H. pylori*-negative population. Furthermore, mtDNA mutations were accumulated more in haplogroup B than in that of D.

**Figure 7:**
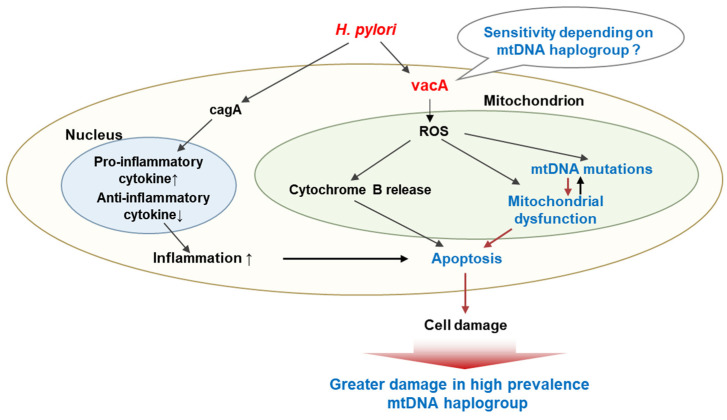
The putative mechanism between the mtDNA haplogroup and the prevalence of *H. pylori*. The high prevalence of mtDNA haplogroup could be susceptible to virulent factors of *H. pylori*, which could induce the accumulation of mtDNA mutations, mitochondrial dysfunction, and apoptosis, resulting in greater cellular damage.

This study demonstrated that the mtDNA haplogroup was associated with cellular damage upon *H. pylori* infection (Figure 7). The high prevalence of mtDNA haplogroup could be susceptible to virulent factors of *H. pylori*, which could induce greater cell damage due to increased mitochondrial dysfunction and apoptosis. Further, these haplogroup mtDNA mutations could be accumulated mtDNA mutations frequently.

## 4. Discussion

In this study, we investigated the association of mitochondrial haplotypes with the prevalence of *H. pylori* and damage by *H. pylori* infection. Firstly, we found haplotype B showed higher frequency in *H. pylori* patients than in that of nonpatients, and we demonstrated that haplogroup B showed susceptibility to cell damage by *H. pylori* infection using assessment of apoptosis and mitochondrial dysfunction. Previous studies reported the induction of apoptosis and mitochondrial dysfunction by *H. pylori* infection; however, haplogroup was not considered [28,29,30], which is different from our study.

Furthermore, mtDNA haplogroup B revealed more apoptosis and reduced mitochondrial respiration upon *H. pylori* infection than another haplogroup, implying that there is also a diversity of susceptibility to the damage caused by *H. pylori* infection, depending on the mtDNA haplogroup.

Many studies showed that genetic polymorphism may affect susceptibility to *H. pylori* infection or related diseases [11,14,31]. Therefore, we also hypothesized that the haplogroup of mtDNA could be associated with susceptibility to *H. pylori*. The distribution of mtDNA haplogroup in the *H. pylori*-negative population in this study was comparable to that in the Korean population reported previously [22], which could support the robustness of the classification of haplogroup using their frequency in the population. In our results, haplogroup B showed a higher frequency in the *H. pylori*-positive population than in that of the *H. pylori*-negative population, and the Korean population and was classified as haplogroup with high susceptibility to *H. pylori* infection. Furthermore, mtDNA mutations accumulated more in haplogroup B than in the low-frequency haplogroup.

We expected that haplogroup B would induce more defects at the cellular level under *H. pylori* infection. According to previous studies, *H. pylori* infection induces apoptosis in gastric epithelial cells, which results in the loss of mucosal integrity and gastric glands, leading to gastric erosion, ulceration, and gastric atrophy [28,29,30,32]. As in these reports, we also demonstrated that *H. pylori* infection-induced apoptosis. However, the level of *H. pylori* infection appeared to be critical to evaluate different aspects of apoptosis depending on the mtDNA haplogroup. Upon *H. pylori* infection at 50 and 100 MOI, the rates of apoptosis were similar among the haplogroup; however, upon *H. pylori* infection at an MOI above 100, there was a higher rate of apoptosis in the high-susceptibility haplogroup than in that of the low one. Therefore, we concluded that *H. pylori* infection at high MOI is suitable to evaluate differences in apoptosis depending on the mtDNA haplogroup, and a haplogroup with a higher frequency in the *H. pylori*-positive population displayed a higher rate of apoptosis, suggesting high-susceptibility to *H. pylori* activity.

Mitochondrial dysfunction was also previously reported to be induced by *H. pylori* infection [19]. *H. pylori* infection reduced oxidative phosphorylation and activity of the electron transport chain in gastric cells [1]. Furthermore, *H. pylori* caused the production of intracellular ROS, which could be an important cause of mtDNA mutations [9]. In this study, we also demonstrated that mitochondrial respiration was damaged by *H. pylori* infection. *H. pylori* infection at high MOI, which could induce severe defects in cells such as apoptosis, disrupted the measurement of mitochondrial respiration. OCR was measured using extracellular acid derived from cultured cells [33]. *H. pylori* induces mitochondrial damage by secreting a pore-forming toxin (VacA), which leads to acidosis in cells [34,35]. *H. pylori* infection at high MOI could induce hyper acidosis in a culture dish, which would disturb the measurement of mitochondrial respiration. Therefore, the MOI of *H. pylori* was reduced for infecting cells, which enabled mitochondrial respiration to be measured normally, suggesting the need for an optimized MOI to measure mitochondrial function upon *H. pylori* infection. Under these optimized conditions, we demonstrated that mitochondrial dysfunction associated with *H. pylori* infection differed depending on the mtDNA haplogroup. The haplogroup that showed a higher frequency in the *H. pylori*-positive population was revealed to be associated with diminished mitochondrial respiration upon *H. pylori* infection. Meanwhile, the haplogroup with a lower frequency in the *H. pylori*-positive population showed less dysfunction upon *H. pylori* infection, suggesting different severity of mitochondrial dysfunction upon *H. pylori* infection, depending on the mtDNA haplogroup.

*H. pylori* was induced epithelial apoptosis and elevated oxidative stress and oxidative damage to mitochondria in vivo in humans [36,37]. In these studies, the cultured fibroblasts by *H. pylori* infection showed comparable results to that of in vivo infection, such as the induction of apoptosis and mitochondrial dysfunction, suggesting that *H. pylori* infection in vitro could represent the infection in vivo. Once *H. pylori* induces mitochondrial dysfunction and acidosis progresses, apoptosis subsequently occurs [34,38]. The mtDNA haplogroup with a higher frequency in the *H. pylori*-positive population displayed severe mitochondrial dysfunction, which could result in a higher rate of apoptosis upon *H. pylori* infection. Virulence factors of *H. pylori*, such as urease subunit A (UreA), cytotoxin-associated gene A protein (CagA), and lipopolysaccharide (LPS), can induce cell apoptosis and increase oxidative stress [23]. *H. pylori*-infected cells could be induced apoptosis and decreased mitochondrial function by those of virulence factors of *H. pylori*.

However, fibroblasts used for the functional study were not genetically identical except for the mtDNA haplogroup, which could affect susceptibility to *H. pylori* infection [11,14]. Therefore, different susceptibility to damage due to nuclear factors should not be ignored.

To establish the chronic infection of the host, bacteria should modulate the immune system of the host, which could enhance resistance and virulence in response to each other between hosts and pathogens [39]. *H. pylori* could have a system to control the host immune response and protect the host against autoimmune diseases, asthma, and esophageal adenocarcinoma, which could also be considered a potential factor of cell cellular damage in the result of our study.

We investigated the potential associations of *H. pylori* with mtDNA mutations and individual mtDNA haplogroup. This study revealed that the number of mtDNA mutations was higher in *H. pylori*-positive gastric tissue than in that of the *H. pylori*-negative one in old age, and that mtDNA mutations were more frequently distributed in functional regions in the *H. pylori*-positive population. mtDNA mutations were not related to sex, whereas the antrum of the stomach tended to accumulate more mtDNA mutations in the *H. pylori*-positive population, but this was not the case in the body of the stomach. These results imply that mtDNA mutations are associated with the prevalence of *H. pylori* infection and that different anatomical positions within the stomach have different levels of susceptibility to *H. pylori* infection [10,40].

*H. pylori* inhabits the mucosal epithelium of the stomach, causing unnoticed chronic gastritis in all carriers, and severe gastric diseases, such as peptic ulcer disease, gastric adenocarcinoma, and mucosa-associated lymphoid tissue lymphoma, in 10–15% of infected individuals [41]. *H. pylori* infection causes DNA damage such as oxidized bases, and single- and double-strand breaks [42]. The instability of mtDNA is also affected by *H. pylori* infection and mtDNA mutations accumulated in *H. pylori*-positive patients [1,9,10]. This phenomenon was also observed in our study. We compared mtDNA mutations in *H. pylori*-positive and -negative populations with the results showing that the number of mtDNA mutations was higher in *H. pylori*-positive patients at the age of 60 years or older, with such mutations being particularly located in functional regions (coding regions and RNA genes), which could constitute evidence that *H. pylori* affects mitochondrial function. The number of mtDNA mutations under 15% heteroplasmy was increased and heteroplasmy was increased in mtDNA mutations above 15% heteroplasmy by *H. pylori* infection, suggesting that *H. pylori* is an important factor inducing mtDNA mutations potentially.

mtDNA mutations showed no sex bias within the *H. pylori*-positive population, but the biopsy positions of the stomach appeared to be associated with the accumulation of mtDNA mutations. The antrum harbored more mtDNA mutations after *H. pylori* infection compared with that of the body of the stomach. This could be caused by the higher distribution of *H. pylori* or a stronger inflammatory reaction in the antrum upon infection with *H. pylori* [40]. Regarding the distribution of mtDNA mutations in the *H. pylori*-positive population, more mtDNA mutations in this group were nonsynonymous substitutions in protein-coding regions or located in RNA genes than those in the *H. pylori*-negative population, suggesting the association of *H. pylori* with the abnormal function of mitochondria.

Previous studies reported the accumulation of mtDNA mutation by *H. pylori* infection, but there was no consideration of haplogroup in *H. pylori* patients [1,9,10]. We demonstrated a higher accumulation of mtDNA mutations in high-frequency haplogroup in the *H. pylori*-positive population, which could support understanding the association between the prevalence of *H. pylori* and mtDNA haplogroup.

*H. pylori* impair mitochondrial DNA repair mechanisms lead to gastric epithelial cells being susceptible to the accumulation of mitochondrial DNA instability, which contributes to gastric carcinogenesis [1,10]. Further, *H. pylori* affects mitochondrial DNA replication [43]. Haplogroup B has accumulated more mtDNA mutations in this study, suggesting that this haplogroup could show severely impaired DNA repair or replication mechanism than other haplogroups.

Although this study focused only on mtDNA mutation associated with *H. pylori*, however, other factors, such as epigenetic factors, could be complementary with mtDNA mutation for the prevalence or pathogenesis in *H. pylori* [44]. Genetics and epigenetics are complementary to human disease [45], and therefore, we could also consider mtDNA mutations and epigenetics together to define the etiology of *H. pylori* injection.

Further, the accumulation of mtDNA mutations in *H. pylori* patients could be related to the modulated immune system by the bacterial pathogen [39], which also be considered a crucial factor for mtDNA integrity.

For 28 *H. pylori*-positive subjects, each *H. pylori* subject comes from a different environment and family, and therefore, we speculated on the possibility of different and diverse strains in each dividual [39]. A previous study reported that a high level of genetic *H. pylori* heterogeneity would explain the low heritability and difficulty in replicating the results of *H. pylori* association studies [39,46], which could a potential hurdle to confirm our claim in further study.

For a deeper insight into the impact of mtDNA haplogroup upon *H. pylori* infection, further studies are needed to confirm the association between *H. pylori* and mtDNA haplogroups with the large-scale screening of mtDNA haplogroup in *H. pylori* patients. Further, if the finding of a high frequency of haplogroup in *H. pylori*-infected patients is replicated in other populations, it would provide powerful evidence for the association between *H. pylori* infection and mtDNA haplogroup, and the prevalence of *H. pylori* in genetically sensitive individuals could be reduced by introducing preventive strategies to minimize environmental causes.

## 5. Conclusions

Our study demonstrated that mtDNA haplogroup B was present at a high frequency in the *H. pylori*-positive population, resulting from higher susceptibility to *H. pylori*. This haplogroup was associated with greater damage to mitochondrial function and higher apoptosis upon *H. pylori* infection. However, there were limitations, as this was a pilot study with small sample size and the use of fibroblasts and a commercial *H. pylori* strain. Further studies on virulence factors of *H. pylori* strains isolated from *H. pylori* patients are required.

## Figures and Tables

**Table 1:** Information on study subjects.

*H. pylori*-Positive Subjects				*H. pylori*-Negative Subjects			
Subject No.	Sex	Age	Mitochondrial Haplotype	Subject No.	Sex	Age	Mitochondrial Haplotype
1	M	45	M8a	1	M	48	G1a
2	M	45	N9a	2	M	54	F1a
3	M	46	B5b	3	M	54	D4i
4	M	49	D4i	4	M	56	M7b
5	F	50	M7c	5	F	57	D4a
6	M	50	D4b	6	M	58	D4g
7	F	51	N9a	7	M	60	D4b
8	M	52	G3a	8	M	60	D4j
9	M	52	B4c	9	M	61	C
10	M	53	F1b	10	F	67	B5b
11	F	54	G3a	11	M	67	G2a
12	M	57	D4a	12	M	68	M7c
13	M	58	B5a	13	M	70	D4f
14	F	59	D4j	14	F	72	B5a
15	F	60	A	15	F	73	M10a
16	M	61	M7b				
17	M	64	N9a				
18	M	64	M7b				
19	M	67	B4c				
20	F	67	M7b				
21	F	67	B5b				
22	M	68	M9a				
23	F	69	D4h				
24	M	72	B4a				
25	F	73	D5a				
26	F	73	F1a				
27	M	78	M10a				
28	M	78	B4b				

**Table 2:** Summary of patients’ information.

Group		No. of *H. pylori*-Positive (Mean Age ± SEM)	No. of *H. pylori*-Negative (Mean Age ± SEM)
Age	<60	14 (52 ± 1)	6 (55 ± 1)
	60≤	14 (69 ± 1)	9 (66 ± 1)
Sex	Male	18 (59 ± 3)	11 (60 ± 2)
	Female	10 (62 ± 3)	4 (67 ± 4)

## Data Availability

The data presented in this study are available in the article and its Supplementary Material.

## Supplementary Materials

The followings are available online at https://www.mdpi.com/article/10.3390/cells10092482/s1, Table S1: The total mtDNA variants detected in the stomach of *H. pylori*-positive subjects, Table S2: The total mtDNA variants detected in the stomach of *H. pylori*-negative subjects, Table S3: The summary of mtDNA mutations detected in the stomach.
